# Supplementary material for: Assessment of technical and clinical utility of a bead-based flow cytometry platform for multiparametric phenotyping of CNS-derived extracellular vesicles
Source: Cell Commun Signal. 2023 Oct 6;21:276. doi: 10.1186/s12964-023-01308-9 (PMC10559539; doi:10.1186/s12964-023-01308-9)
Supplement: Supplementary file 3 — Additional file 2. [file 12964_2023_1308_MOESM2_ESM.docx]

**Original, uncropped blots for Figure 2 b:**


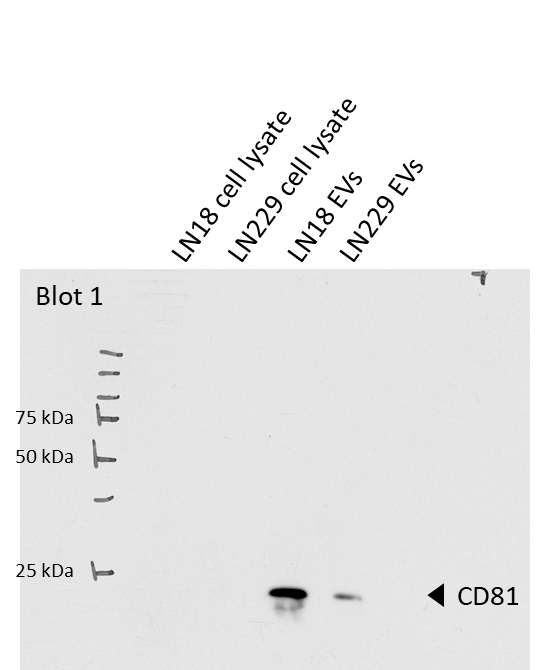


Blot 1.1: Original blot and staining of bands shown for CD81 in LN18 and LN229 EVs, exposure time: 5 s.


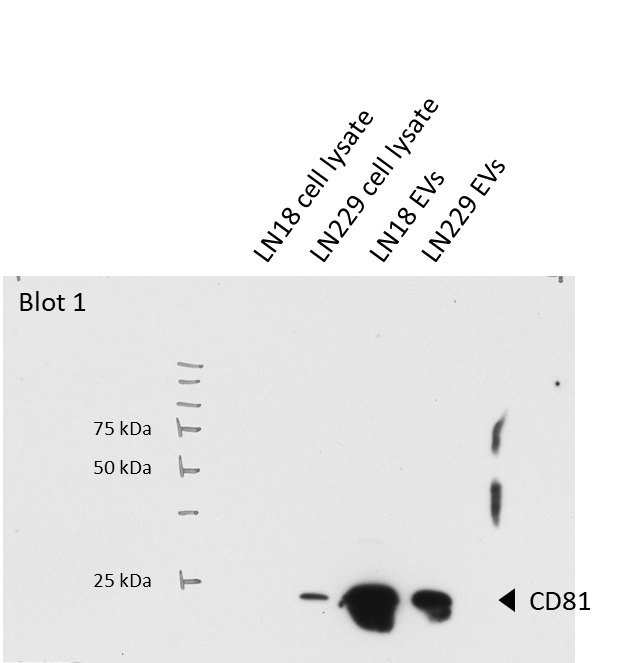


Blot 1.2: Original blot and staining of bands shown for CD81 in LN18 and LN229 cell lysates, exposure time: 2 min.


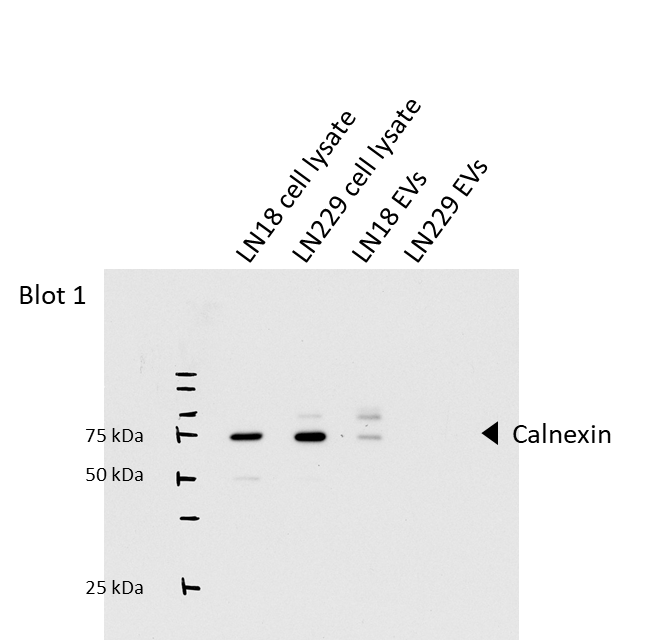


Blot 1.3: Original blot and staining of bands shown for Calnexin in LN18 and LN229 cell lysates and EVs, exposure time: 5 s.


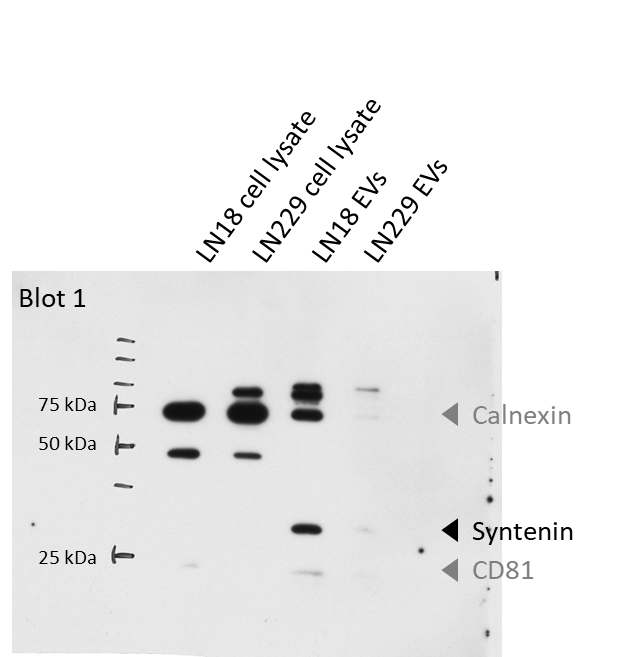


Blot 1.4: Original blot and staining of bands shown for Syntenin in LN18 and LN229 cell lysates and EVs, exposure time: 2 min.


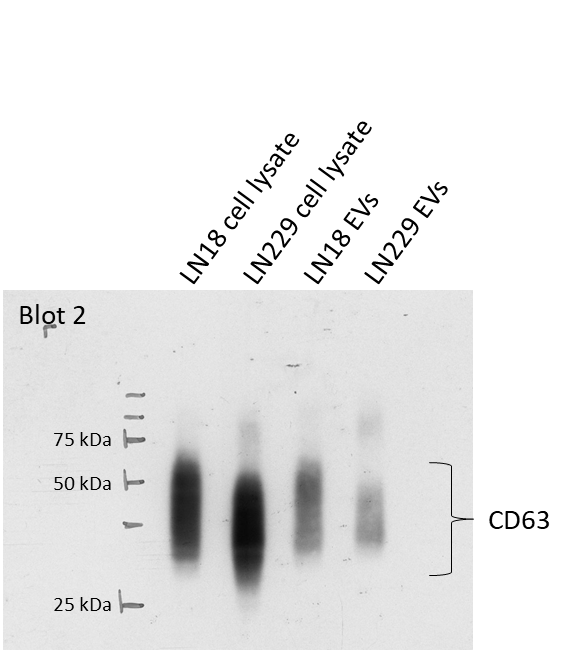


Blot 2.1: Original blot and staining of bands shown for CD63 in LN18 and LN229 cell lysates and EVs, exposure time: 5 s.


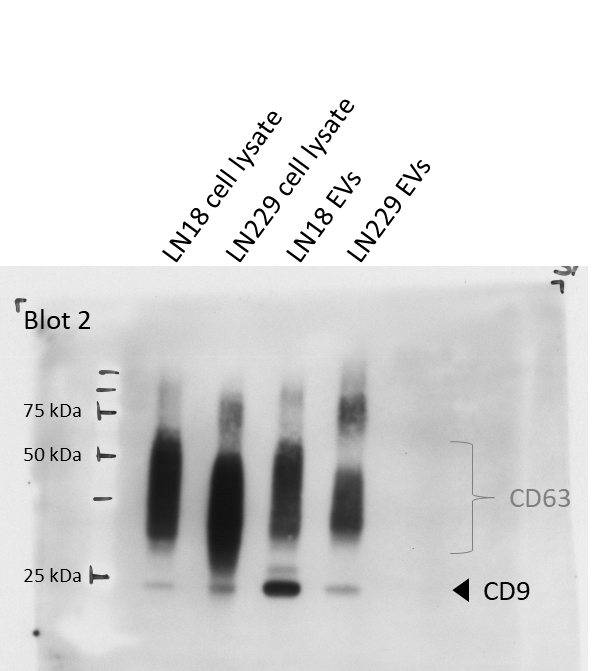


Blot 2.2: Original blot and staining of bands shown for CD9 in LN18 and LN229 cell lysates and EVs, exposure time: 3 s.


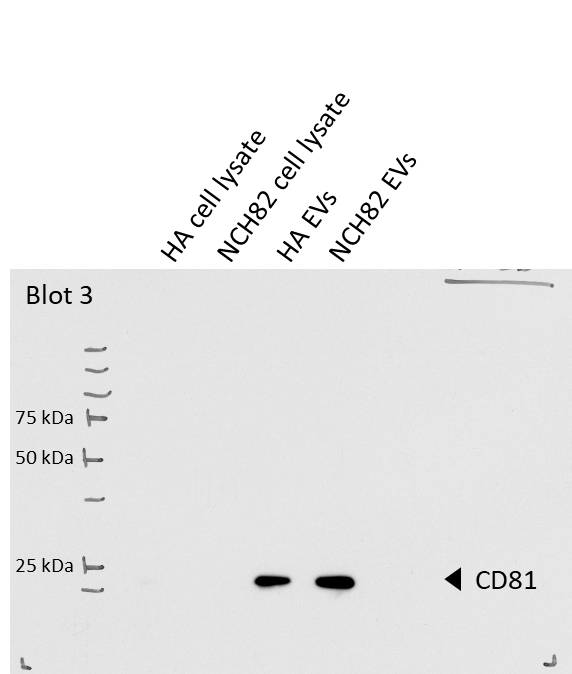


Blot 3.1: Original blot and staining of bands shown for CD81 in HA and NCH82 EVs, exposure time: 5 s.


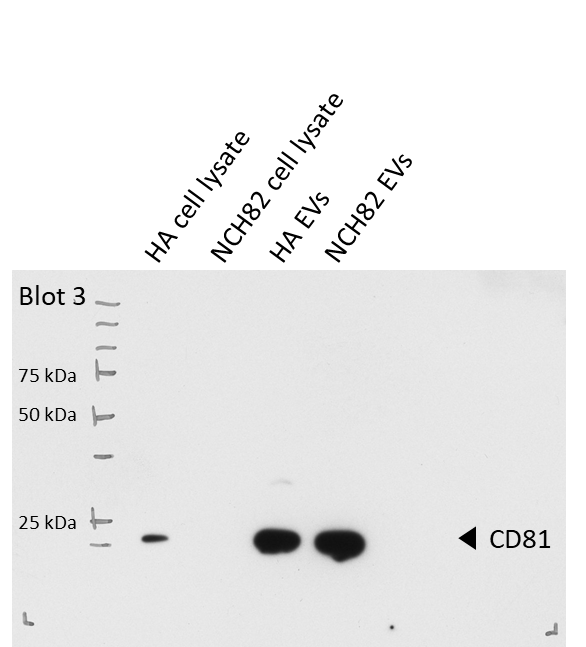


Blot 3.2: Original blot and staining of bands shown for CD81 in HA and NCH82 cell lysates, exposure time: 2 min.


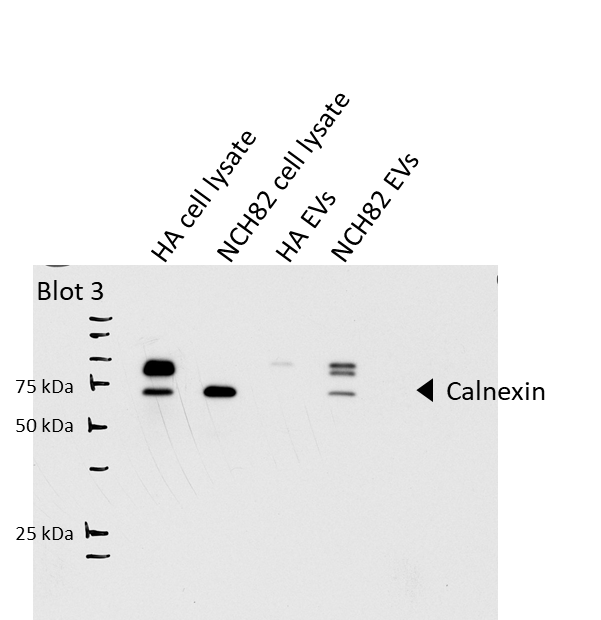


Blot 3.3: Original blot and staining of bands shown for Calnexin in HA and NCH82 cell lysates and EVs, exposure time: 10 s.


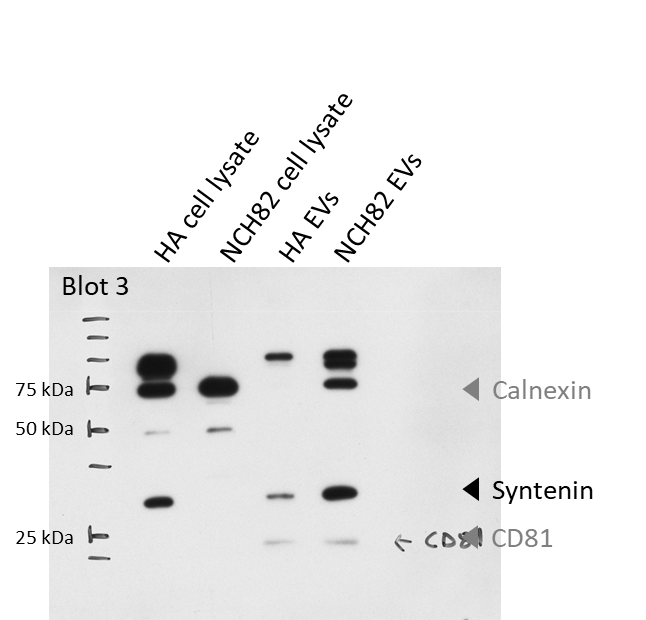


Blot 3.4: Original blot and staining of bands shown for Syntenin in HA and NCH82 cell lysates and EVs, exposure time: 2 min


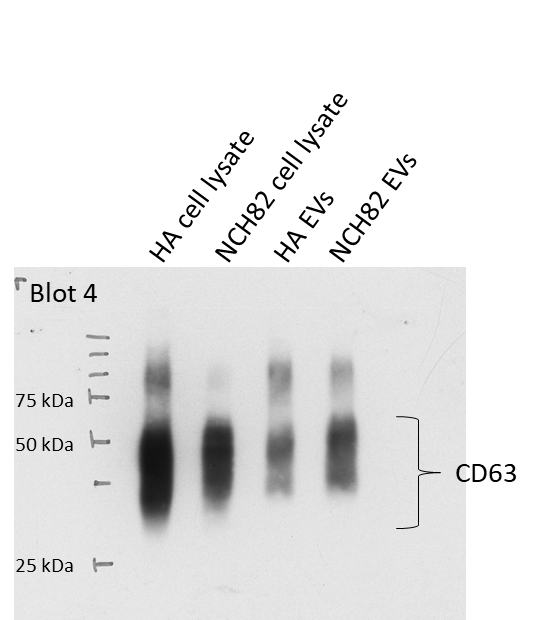


Blot 4.1: Original blot and staining of bands shown for CD63 in HA and NCH82 cell lysates and EVs, exposure time: 5 s.


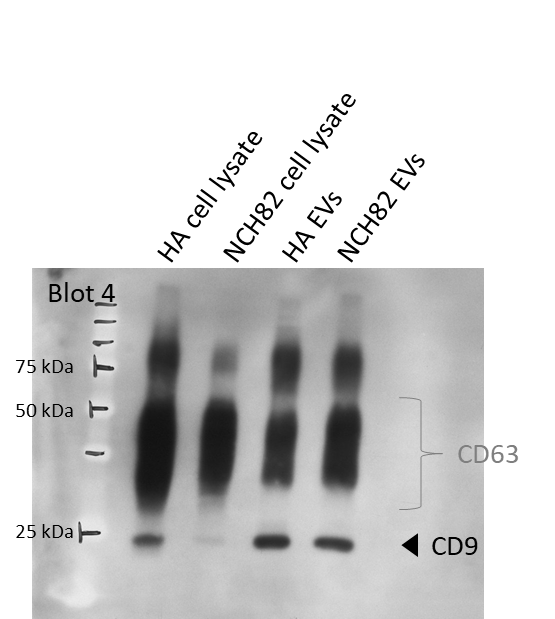


Blot 4.2: Original blot and staining of bands shown for CD9 in HA and NCH82 cell lysates and EVs, exposure time: 5 s.
